# Gap Analysis of Priority Medicinal Plant Species in the Kingdom of Saudi Arabia

**DOI:** 10.3390/plants14172629

**Published:** 2025-08-23

**Authors:** Ibrahim Jamaan Alzahrani, Joana Magos Brehm, Nigel Maxted

**Affiliations:** 1 School of Biosciences, University of Birmingham, Birmingham B15 2TT, UK; ijalzahrani@bu.edu.sa (I.J.A.); joanabrehm@gmail.com (J.M.B.); 2 Department of Biology, Faculty of Science, Al-Baha University, Al-Baha 65779, Saudi Arabia

**Keywords:** medicinal plants, ecogeographic land characterization, in situ and ex situ conservation, complementarity analysis, Saudi Arabia

## Abstract

Medicinal plant species are crucial biological resources, and yet their conservation in the Kingdom of Saudi Arabia remains insufficiently studied. This study conducts a comprehensive gap analysis of 74 priority medicinal plant species in the Kingdom of Saudi Arabia to assess their spatial distribution, identify conservation gaps and propose strategic recommendations. Occurrence records were collected from field surveys and global biodiversity databases, followed by ecogeographical land characterization and conservation gap analyses using the CAPFITOGEN3 tools. The results reveal significant disparities in in situ and ex situ conservation efforts, with two biodiversity hotspots, Asir and Jazan, containing the highest species diversity. While 66 species occur within protected areas, seven species are currently only recorded outside protected areas, indicating opportunities for expanding conservation efforts. Complementarity analysis identified 13 optimal protected areas for priority medicinal plants’ conservation, alongside 20 potential sites outside protected areas that could serve as other effective area-based conservation measures. Ex situ conservation remains critically limited for many species, with only 10 represented in genebanks and all accessions currently stored internationally, although some medicinal plant species may have broader global distributions. To bring about improved outcomes of conservation, the expansion of in situ conservation coverage, integration of other effective area-based conservation measures, strengthening of national genebanks and leverage of biotechnology and geospatial tools is recommended by this study. The findings of this study can be used to develop a more systematic and sustainable approach to the conservation of medicinal plants in the Kingdom of Saudi Arabia.

## 1. Introduction

For millennia, humans have depended on plants for sustenance, housing, energy, textiles, attire, medicines and so on [1]. Many ancient traditional medicine systems are based on plants [2]. Medicinal plants (MPs) are used as models for designing synthetic drugs, are collected from the wild or cultivated and then consumed directly, and are effective in treating various illnesses, particularly in traditional medicine [3]. Out of the 390,900 flowering plant species found worldwide [4], more than 50,000 are used for medicinal purposes [5]. The World Health Organization states that approximately 80% of the world’s population depends on herbal medicine for primary healthcare [6,7], particularly those based on subsistence cultures or residing in isolated mountainous regions [8]. The demand for herbal medicines continues to grow in developed countries, where many people view them as safe, effective and relatively affordable [9,10,11]. However, approximately 15,000 medicinal plant species globally face extinction due to overexploitation, habitat loss and climate change [12,13]. Economically, many societies rely heavily on medicinal plants and their derivatives. Global exports of medicinal plants reached approximately USD 3.6 billion in 2014 [14] and are anticipated to reach USD 5 trillion by 2050 [15].

The Kingdom of Saudi Arabia (KSA) is one of the most biologically diverse countries of the Arabian Peninsula [16,17]. Its flora comprises around 2250 species from 142 families [16,17,18]. The greatest species diversity is found in the mountainous northwestern and southwestern regions, accounting for 70% of the country’s plant species [18]. Of these, over 1200 species have medicinal value [19,20].

Despite this rich biodiversity, the Kingdom of Saudi Arabia’s flora faces numerous threats, including extreme weather events, heavy metal contamination, salinity, altered soil pH, drought and extreme temperatures, many of which are associated with climate change [21]. Overharvesting, urbanisation, overgrazing and human environmental mismanagement are also factors contributing to the decline of plant biodiversity and the increasing vulnerability of medicinal plant species [11,22]. Moreover, the lack of legislative or regulatory oversight regarding the wild harvesting, production and use of herbal medicines further threatens the country’s medicinal plants [23]. Active conservation and an increased cultivation of these species is urgently needed to ensure their survival in the long-term [24].

There are two main strategies for conserving biodiversity: in situ and ex situ conservation. The Convention on Biological Diversity (CBD) in 1992 defines “in situ conservation” as “the conservation of ecosystems and natural habitats and the maintenance and recovery of viable populations of species in their natural surroundings and, in the case of domesticated or cultivated species, in the surroundings where they have developed their distinctive properties”. In the same text, “ex situ conservation” is defined as “the conservation of components of biological diversity outside their natural habitats” [25,26]. In the Kingdom of Saudi Arabia, efforts to conserve biodiversity are relatively recent and face several challenges, such as limited resources, a lack of legislative frameworks and public awareness. Additionally, biodiversity itself is threatened by factors including climate change and invasive species [27]. One of the global challenges in conservation is identifying regions with the highest biodiversity and determining how to prioritise limited resources for conservation effectively and efficiently [28]. In recent years, researchers in the Arabian Peninsula have focused on identifying biodiversity hotspots and addressing conservation gaps for endemic and endangered medicinal plants [29]. As a result, protected areas were established to safeguard biodiversity from degradation [30], with the aim of actively managing and monitoring target species within these areas [31]. The Kingdom of Saudi Arabia contains 79 protected areas [32], with most of them located in the northwestern and southwestern regions of the country. This is largely due to the rich biodiversity found in these areas [18].

Gap analysis is a widely used method for assessing biodiversity conservation. It helps identify species or habitats that are inadequately represented in current conservation efforts, whether at the local, national, or global level, and considers both in situ and ex situ approaches [31,33,34]. This method has become increasingly utilised for conservation planning [35]. The basic process of gap analysis consists of four steps [31]: (a) identifying the target taxa and area, (b) evaluating the ecogeographic and genetic diversity within the target taxa, (c) identifying conservation gaps and (d) reformulating conservation strategies. Gap analysis for plant conservation has been applied to crop wild relatives (CWR) [36,37,38,39], threatened species [40,41] and medicinal plants [29,42,43].

Based on the priority checklist of medicinal plants for the Kingdom of Saudi Arabia defined by Alzahrani et al. [44], this study aims to evaluate the diversity of priority medicinal plants in the Kingdom of Saudi Arabia, investigate diverse options for conservation application and provide recommendations for conservation implementation. Specifically, it seeks to (i) evaluate the spatial distribution of priority medicinal plant species in the Kingdom of Saudi Arabia, (ii) identify sites where in situ conservation of these species could occur, (iii) evaluate the current status of ex situ collections and identify species that require further collecting and (iv) propose effective conservation strategies for the sustainable management of these valuable genetic resources.

## 2. Results

### 2.1. Overview of Data and Distribution of Medicinal Plants in the Kingdom of Saudi Arabia

**Figure 1:**
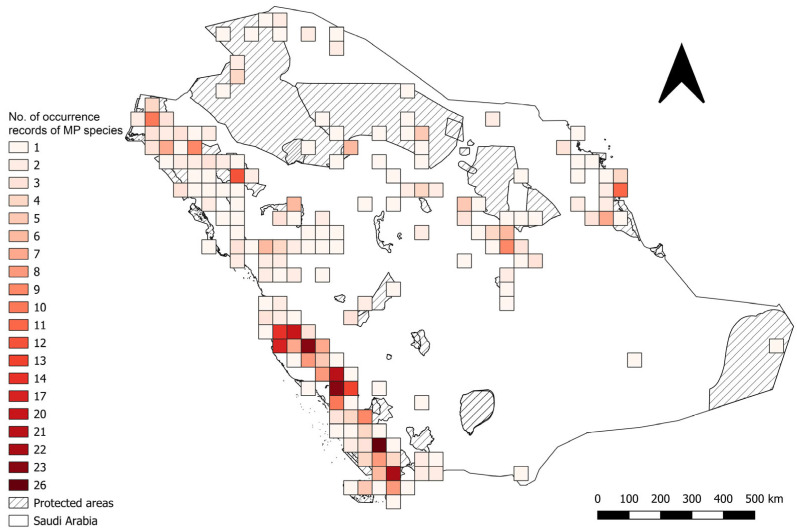
Number of species occurrence records of 73 medicinal plant species in the Kingdom of Saudi Arabia.

**Figure 2:**
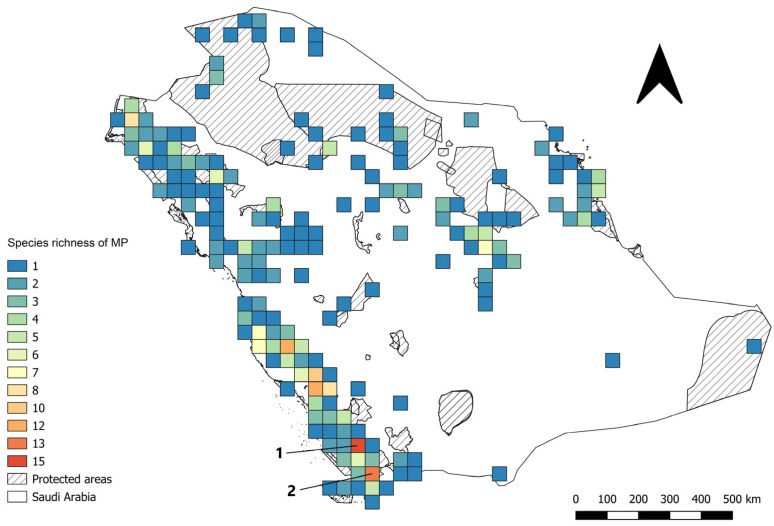
Medicinal plant species richness in two hotspot areas: (1) Asir and (2) Jazan Provinces in the southern region of Saudi Arabia, which contain the highest number of priority species.

Out of the 1099 species occurrence records compiled from the Kingdom of Saudi Arabia (Table S1), a total of 1003 (91%) were considered valid after carrying out data quality checks. These viable records represented 73 of the 74 priority medicinal plants (Figure S1 and Table S1). The number of presence records per species varied between 1 and 76. *Olea europaea* L. subsp. *cuspidata* (Wall. & G.Don) Cif. (76), *Senna italica* Mill. (73) and *Portulaca oleracea* L. (46) showed the highest occurrence counts, whereas *Glycyrrhiza glabra* L. had only one record (Table S1). The national distribution of records showed a concentration in the western and southwestern regions, with 576 records (57%), with progressively fewer records in the northern and northwestern regions (256 records, 26%), the central region (108, 11%) and the eastern region (63, 6%) (Figure 1). Asir and Jazan, both located in the southwestern region (Figure 2), were identified as target medicinal plants hotspots. The Imam Faisal bin Turki Royal Reserve, situated within these hotspots, exhibited the highest species richness, with 40 medicinal plant species recorded (Table S2).

### 2.2. Ecogeographic Land Characterization

**Figure 3:**
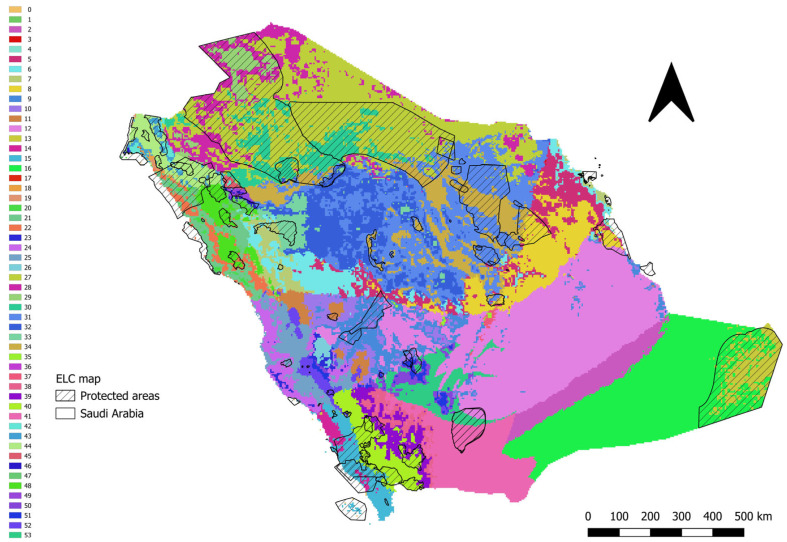
ELC map for the Kingdom of Saudi Arabia identifying 52 ecogeographically distinct zones.

A generalist ELC map identifying 52 ELC zones for priority medicinal plants in the Kingdom of Saudi Arabia was developed using 12 ecogeographic variables (four bioclimatic, four edaphic and four geophysical variables) (Figure 3 and Table S3). Out of the 52 ELC zones, 41 had occurrence records, and 31 of these included records within protected areas. Priority medicinal plants were unevenly distributed across the ELC zones, with zones 52, 15 and 40 having the highest numbers of occurrence records—101, 114 and 181 records, respectively (Table S4). In addition, these same ELC zones also had the highest species richness, hosting the greatest number of different medicinal plant taxa. Meanwhile, the largest number of medicinal plant populations within protected areas was observed in ELC zones 44, 15 and 40, whereas the highest number outside protected areas was found in ELC zones 40, 25 and 52 (Figure S2 and Table S5). The taxa that occur in the most varied ecogeographic environments (i.e., ELC zones) were *Solenostemma arghel* (Delile) Hayne, *Cistanche tubulosa* (Schenk) Wight ex Hook.f., *O. europaea* subsp. *cuspidata* and *Teucrium capitatum* L. In contrast, *Allophylus rubifolius* (Hochst. ex A.Rich.) Engl., *Dianthus deserti* Kotschy, *G. glabra*, *Sonchus asper* (L.) Hill and *Thymus decussatus* Benth. were confined to a single ELC zone (Table S6).

### 2.3. In Situ Gap Analysis

The analysis found 66 of the 73 taxa were represented by 464 georeferenced species presence records within the protected areas of the Kingdom of Saudi Arabia. These were spread across 45 reserves, with the reserves having the highest number of records and taxa being the Imam Faisal bin Turki Royal Reserve (143 records, 40 species), Asir National Park (55 records, 24 species), Prince Mohammed bin Salman Royal Reserve (34 records, 15 species), King Salman Bin Abdalaziz Royal Natural Reserve (25 records, 13 species), Jabal Shada Al-A’la (20 records, 12 species) and Tuwaiq Reserve (14 records, 12 species) (Table S2). In addition, the taxa with the highest number of occurrence records within protected areas was *Ficus palmata* Forssk. (46 records), *O. europaea* subsp. *cuspidata* (34), *Vachellia origena* (Hunde) Kyal. & Boatwr. (31), and *S. italica* (29) (Table S7). On the other hand, seven species were only recorded outside existing protected areas: *Senna holosericea* (Fresen.) Greuter (six records), *Eruca sativa* Mill. and *Balanites aegyptiaca* (L.) Delile (three records each), *Scorpiurus muricatus* L. and *A. rubifolius* (two records each) and *G. glabra* (one record) (Table S8).

### 2.4. Complementarity Analysis

**Figure 4:**
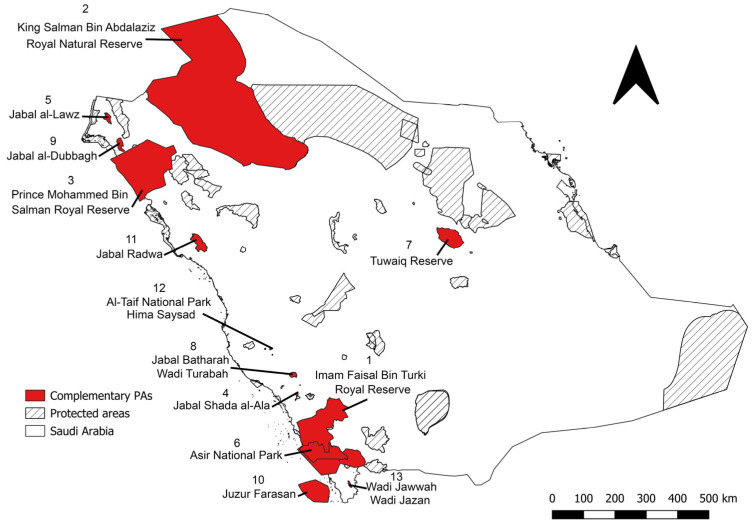
Thirteen complementary protected areas (PAs) were identified for medicinal plant species found in the Kingdom of Saudi Arabia. The numbers represent the ranking of these protected areas.

The complementarity analysis identified 13 priority protected areas for the conservation of 64 of the 74 priority medicinal plants (Figure 4, Table 1), representing a total of 268 populations. The Imam Faisal bin Turki Royal Reserve has the highest diversity and number of recorded species, with 35 species and 133 records. It is followed by the King Salman Bin Abdalaziz Royal Natural Reserve, which contains a total of 11 different species and 25 occurrence records, and the Prince Mohammed bin Salman Royal Reserve, which includes 4 species and 32 records (Table 1). The species with the highest population records in these complementary protected areas was *F. palmata*, with 29 records, followed by *O. europaea* subsp. *cuspidata* and *V. origena*, each with 21 records (Table S9).

**Figure 5:**
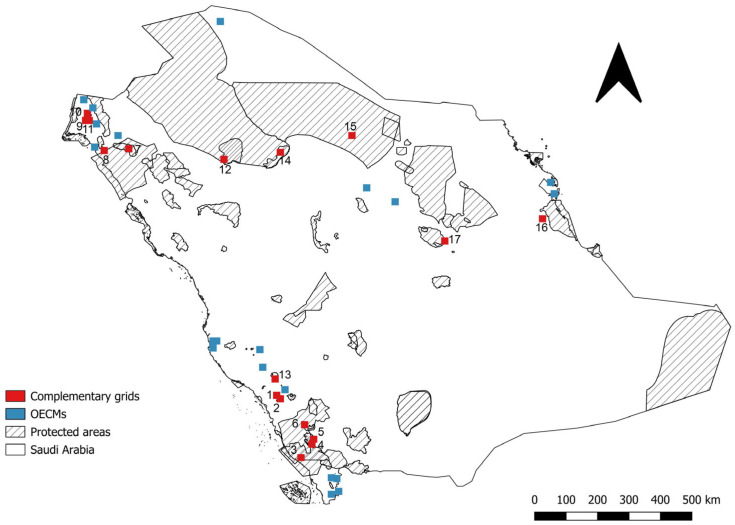
Complementary sites for medicinal plant species found in the Kingdom of Saudi Arabia were identified using 5 × 5 km grid cells; seventeen sites (red) overlap with existing protected areas and twenty (blue) are located outside protected areas, making them potential sites for novel protected areas or other effective area-based conservation measures implementation.

Additionally, 37 complementary sites, identified based on grid cells, were selected to support the conservation of medicinal plant species (Figure 5). Species richness varied across these grid cells, with the most diverse cell containing nine species (Table S10). Of these 37 sites, 17 are located within existing protected areas, while 20 are located outside protected areas boundaries. These external sites were identified based on overall species richness but were not exclusively selected for species absent from existing protected areas. Therefore, while they represent important areas of medicinal plant diversity, further analysis is recommended to prioritise grid cells that contribute unique taxonomic or ecogeographic diversity not currently conserved within the protected areas network. These external sites could be considered for implementing other effective area-based conservation measures to enhance the conservation of unprotected medicinal plant diversity (Figure 5). A total of 49 species were found within the 17 existing protected areas. The protected area with the highest species diversity was Jabal Shada al-A‘la with nine species. It was followed by the extension to Jabal Shada with six species. Next were Asir National Park, Wadi Tayyah, Raydah and Wadi ‘Iya’ / Ballasmar, each with three species. Meanwhile, the remaining ten protected areas each contained two species (Table S11).

### 2.5. Ex Situ Gap Analysis

There were 10 priority species (14%) with existing ex situ accessions, represented by a total of 15 accessions recorded in genebanks (Table S12), whereas the remaining 63 species (86%) had no recorded ex situ accessions based on the data used. All 10 species with ex situ accessions were underrepresented in terms of sampling effort and ecogeographic diversity, with fewer than five samples each, which is below the minimum recommended threshold for population conservation (Table S12) [45]. Moreover, all of these samples were stored only in international genebanks, specifically in the Millennium Seed Bank. The species with the highest number of accessions was *Phoenix dactylifera* L. (four accessions), followed by *Umbilicus rupestris* (Salisb.) Dandy and *V. origena*, each with two accessions, while the remaining seven species had only one accession each (Table S12). Given that the ecogeographic diversity analysis revealed that only 8 out of 52 ELC zones (15%) were represented in genebanks and they are associated with distinct ecogeographic zones, then each is expected to be correlated with patterns of genetic diversity, so it is likely the few medicinal plant accessions conserved ex situ reflect poor taxonomic and genetic coverage of the Kingdom of Saudi Arabia’s medicinal plant diversity. Among the zones with medicinal plant samples in genebanks, zones 40 and 52 were the most frequently recorded, each appearing twice (Table S13). However, the ecogeographic diversity analysis revealed that most species’ ex situ accessions covered very few or no ELC zones, indicating a poor representation of their adaptive diversity across the existing genebank collections.

## 3. Discussion

One of the most biodiverse regions in the Arabian Peninsula, the Kingdom of Saudi Arabia hosts a wealth of flora [16,17]. However, overexploitation, habitat loss and climate change put this rich biodiversity resource at risk [11,22]. Both in situ and ex situ conservation gaps must be identified urgently as part of conservation planning and to ensure this critical resource is maintained for immediate and future utilization [35]. The present study reports a gap analysis of priority medicinal plant species in the Kingdom of Saudi Arabia, using both in situ and ex situ conservation methodologies. The findings point out critical weaknesses in current medicinal plant conservation. They make actionable recommendations for improving the conservation and sustainable utilisation of these valuable species.

The analysis of 73 out of 74 priority medicinal plant species in the Kingdom of Saudi Arabia, for which occurrence data are available, revealed significant gaps in conservation efforts. The study highlights substantial variation in the number of occurrence records among taxa, likely reflecting differences in species distribution, ecological preferences and collection effort. The prominence of *Olea europaea* subsp. *cuspidata* aligns with its known adaptability to semi-arid and meso-humid environments [46], contributing to its widespread presence in the country. Similarly, *Senna italica*, commonly used in traditional medicine for its laxative properties, is naturally abundant in dry and desert regions [47].

Two biodiversity hotspots with the highest diversity of medicinal plants were identified, both located in the southwestern region of the Kingdom of Saudi Arabia. According to Osman et al. [18], this region is one of the largest areas of biodiversity in the country. A noticeable observational bias was observed in the Asir and Jazan regions. The bias towards certain areas over others can be explained by their ease of access, which in turn leads to a higher frequency of sample collection by botanists [48] in addition to their proximity to researchers’ residences [49]. Furthermore, the challenging terrain of the Arabian Peninsula, including its mountainous and desert regions, makes access to these areas difficult, which has led to their underrepresentation in survey efforts and, consequently, in conservation planning [50]. Thus, this does not reflect the true diversity in the country and further surveying of medicinal plants in the Kingdom of Saudi Arabia is a critical priority. It is essential to address these biases, and future collection efforts should focus on under-sampled regions and diverse habitats to ensure more accurate and representative occurrence records [51].

The in situ gap analyses identified 66 medicinal plant taxa within protected areas, but these are primarily subject to passive conservation, lacking active management, monitoring or intervention [52,53]. Notably, seven priority medicinal plants were completely absent from existing protected areas, indicating an urgent need for active conservation. This trend of passive conservation requiring supplementary efforts was also reported in studies on CWR conservation in Norway [54] and medicinal plant conservation in Indonesia [42].

The results indicate that the species found within protected areas were documented in 45 out of 79 existing protected areas. The King Salman bin Abdulaziz Royal Natural Reserve is the largest in the country in terms of area, covering approximately 130,700 km^2^ However, this study indicates that the Imam Faisal bin Turki Royal Reserve, which covers approximately 30,153 km^2^, contains the highest number of priority medicinal plants, with a total of 40 species. A key ongoing challenge within these protected areas is the implementation of effective conservation management strategies, particularly in preserving biodiversity [55] and its intra-specific genetic diversity.

Creating a network of complementary reserves is key to the development of a well-represented and effective conservation system. This strategy guarantees the efficient use of land and the protection of a variety of species whilst keeping costs to a minimum [56]. This well-established method is widely used in conservation planning and has been applied to crop wild relatives across several countries and regions, e.g., [39,51,55,57], and it has also been applied to medicinal plants in Indonesia [42]. This study identified 13 complementary protected areas that together conserve the greatest possible diversity of medicinal plants based on taxon and ecogeographic representation. Within these, 17 complementary grid sites were identified, located inside existing protected areas, where the monitoring and management of medicinal plant species would be relatively straightforward and cost-effective, ultimately enhancing the conservation value of these areas [58]. Additionally, 20 complementary grid sites were identified outside existing protected areas. These areas could be prioritised for the establishment of other effective area-based conservation measures or other forms of managed conservation to protect important medicinal plant diversity not currently covered by the formal protected areas network. The expansion of existing reserves, potential establishment of novel protected area or establishment of new other effective area-based conservation measures is thus possible within these 20 locations, providing ground truthing corroborates the modelling predictions of CWR presence. The complementary analysis revealed that many of the complementary protected areas, complementary sites and other effective area-based conservation measures are located in the northwestern and particularly southwestern regions of the Kingdom of Saudi Arabia. This is due to the high biodiversity in these areas [18]. Thus, further surveys are required in these areas to further document medicinal plants and apply this knowledge to actively conserve them in situ and ex situ and so ensure medicinal plants’ long-term taxonomic and genetic diversity sustainability.

The ELC map for medicinal plant taxa in the Kingdom of Saudi Arabia can support the conservation of ecogeographic and potentially adaptive diversity across different environmental zones, serving as a useful surrogate in the absence of detailed genetic data [59]. The ELC mapping tool, developed by Parra-Quijano et al. [59,60], illustrates a number of possible plant adaptation scenarios dependent on ecogeographic diversity which are likely to be correlated with genetic adaptation and unique allele differentiation over time. The ELC map for medicinal plant taxa in the Kingdom of Saudi Arabia shows that 31 out of 52 ELC zones were present in protected areas, while just 8 ELC zones were represented in genebanks; the ecogeographic diversity analysis for each taxon showed that most species had very limited or no coverage of their full ecogeographic variation in ex situ collections. This highlights a critical gap in conserving the adaptive diversity of medicinal plant taxa. Previous research [61,62] emphasised the necessity to prioritise collections from underrepresented zones to capture maximum genetic diversity. Our findings demonstrate the poor genetic conservation coverage of medicinal plants in the Kingdom of Saudi Arabia, reinforce the urgent need for more active conservation and suggest the establishment of a national ex situ conservation strategy for medicinal plant species in the Kingdom of Saudi Arabia, with an emphasis on securing duplicates in both local and international genebanks to enhance long-term preservation efforts [39,63].

Ex situ conservation acts as a complementary approach to preserving biodiversity in situ, making sure that genetic resources continue to represent their natural populations [64]. This method plays a crucial part in preventing species loss and protecting plant diversity for future generations [65]. Its accessibility to germplasm users also facilitates the conservation–utilisation linkage [52,66]. This method is crucial for preventing species loss and safeguarding plant diversity for future generations [65] Various techniques are used in ex situ conservation, including seed storage, genebanks, in vitro preservation through tissue culture and cryopreservation, as well as conservation in botanical gardens, arboretums, pollen storage and DNA storage [52,67]. Ex situ conservation analysis for medicinal plants in the Kingdom of Saudi Arabia revealed that only 10 medicinal plant species (14%) had recorded accessions in genebanks, with all accessions stored exclusively in international genebanks. These findings are based on publicly available global databases and may not reflect undocumented or non-digitized collections, such as those in smaller botanical gardens or national institutions. As such, the identified ex situ conservation gaps reflect gaps in accessible data, not necessarily a complete absence of conservation efforts. All of these species collected in international genebanks have fewer than five populations. According to Brown and Briggs [45], the minimum number of populations required for ex situ conservation is at least five. The lack of national genebank representation for medicinal plant species in the Kingdom of Saudi Arabia highlights a significant conservation gap. Similar challenges were reported in conservation studies on CWR in Norway [54] and Malawi [68], where underrepresentation in genebanks was identified as a major limitation to genetic resource preservation.

Overall, the results of this study underscore the need for integrated conservation strategies that address both in situ and ex situ gaps in medicinal plant species diversity across the Kingdom of Saudi Arabia. While some progress has been made in protecting species and ecogeographic diversity within existing protected areas and genebanks, significant gaps remain, particularly for taxa with limited occurrence records and poor ecogeographic representation. Enhancing data quality, expanding conservation coverage, and fostering national and international collaboration will be essential for ensuring the long-term conservation and sustainable use of medicinal plant genetic resources. The following recommendations outline key actions to address these challenges and are summarized in Table 2.

These recommendations represent a comprehensive and pragmatic conservation framework grounded in scientific evidence, traditional knowledge, and strategic action. To ensure their continued relevance and effectiveness, conservation priorities should be regularly reviewed and updated based on the latest data and research findings. Future prioritization efforts should incorporate not only ecological and geographic criteria but also the cultural and ethnopharmacological significance of medicinal plant species. Particular attention should be given to locally endemic taxa and those with a well-documented history of traditional use in Saudi Arabia, as they represent both biological and cultural assets of national importance.

## 4. Materials and Methods

### 4.1. Species Selection

The 74 priority medicinal plant species investigated in this study were selected following the prioritization framework established by Alzahrani et al. [44]. That study compiled an initial checklist of 1174 medicinal plant taxa occurring in the Kingdom of Saudi Arabia, derived from published literature and online resources, and refined it using a stepwise prioritization process. The criteria applied included known medicinal uses, native status, conservation status based on IUCN categories and Collenette’s expert assessments, and legal use as recognized by the Saudi Food and Drug Authority (SFDA). These criteria align with recommended approaches for medicinal plants and crop wild relative (CWR) conservation [31,34]. The final list of 74 priority medicinal plant species served as the target taxa for the conservation gap analysis presented here.

### 4.2. Occurrence and Diversity of Medicinal Plant Species

Distributional data for 74 priority medicinal plant species (Table S1) in the Kingdom of Saudi Arabia were collected from multiple sources:

(a) Fieldwork (March–August 2024): Field surveys were conducted by the first author in regions identified as medicinal plant hotspots based on herbarium records and relevant literature [16,18,44]. Sites were selected using historical records indicating the presence of priority medicinal plant species. During each site visit, populations of medicinal plants were recorded using handheld GPS devices.
(b) Herbarium records: Data were compiled from the Royal Botanic Gardens, Kew, and the Royal Botanic Garden, Edinburgh (https://data.rbge.org.uk/) (accessed on 17 June 2025) contributing a significant portion of occurrence records.
(c) Biodiversity databases: Additional occurrence records were sourced from the Global Biodiversity Information Facility (GBIF) (http://www.gbif.org) (accessed on 17 June 2025), the BOLD database (http://www.boldsystems.org) (accessed on 17 June 2025) and the Genesys Global Portal on Plant Genetic Resources (https://www.genesys-pgr.org) (accessed on 17 June 2025), providing substantial Supplementary Data.

All occurrence records were compiled using the FAO/Bioversity Multi-Crop Passport Descriptors [70]. Google Maps (https://www.google.com/maps) (accessed on 17 June 2025) was used to georeference specimens with location data that lacked geographic coordinates. To standardise the coordinate format across all records, location records missing decimal degree coordinates were converted into decimal degrees using the Canadensys coordinate conversion tool (https://data.canadensys.net/tools/coordinates) (accessed on 17 June 2025). To maintain data accuracy and prevent duplication, duplicate geographical records referring to the same occurrences, whether sourced from various sources or documented twice from the same source, were removed [71].

The TesTable tool in CAPFITOGEN3 v3.0 was used to verify the occurrence records and make sure they were in an appropriate format, and the GEOQUAL tool was used to assess their quality. In GEOQUAL, the TOTALQUAL100 parameter, which ranges from 0 (lowest quality) to 100 (highest quality), was used as a threshold for data inclusion. Only occurrence records with a TOTALQUAL100 score of 55% or higher were retained for analysis [62].

### 4.3. Ecogeographical Land Characterization Map

There are two types of ecogeographical land characterization (ELC) maps: generalist and species-specific. Generalist ELC maps use ecogeographic variables that represent the various ecogeographic scenarios within a given area. In contrast, species-specific ELC maps focus on variables that are particularly important for defining adaptive ecogeographic scenarios for a single taxon [38,54,55,59,60].

To select the most relevant ecogeographic variables for constructing the generalist ELC map, the SelecVar tool from CAPFITOGEN3 was used. The tool applies correlation and variance analyses to identify variables that maximise environmental diversity representation while minimising redundancy.

Using the ELCmapas tool in CAPFITOGEN3, a generalist ELC map was created to represent the diversity of all taxa in the Kingdom of Saudi Arabia, rather than producing individual ELC maps for each taxon [59]. The final generalist ELC map, generated at a resolution of 5 × 5 km (approximately 2.5 arc minutes), was produced using the Kmeansbic method [62]. This method was chosen because the K-means BIC algorithm effectively identifies the optimal number of ecogeographic groups while balancing model complexity and goodness of fit, making it particularly suitable for ecological and biodiversity data where overfitting must be avoided. It has also been successfully applied in previous conservation studies to accurately delineate ecogeographical zones [61,62].

### 4.4. In Situ Conservation Gap Analysis

In situ conservation gap analysis examines the alignment between the existing diversity within a given area and the portion of that diversity currently protected by active conservation measures [71,72]. For this study, in situ conservation gaps were analysed to identify deficiencies in the conservation of medicinal plants in the Kingdom of Saudi Arabia at both the taxon and ecogeographic levels. This approach aimed to provide comprehension and understanding of the diversity of target taxa within the country.

#### 4.4.1. Taxon-Level Analysis

To identify conservation gaps at the taxon level, medicinal plant occurrences were mapped using QGIS version 3.40 [73]. The PA maps of Kingdom of Saudi Arabia, obtained from the Protected Planet database (https://www.protectedplanet.net/en accessed on 17 June 2025), were consolidated into a unified map covering the entire country. Overlaying the medicinal plant distribution data onto this PA map allowed for the identification of species and occurrences both within and outside these designated areas [68,71]. Additionally, a coverage analysis was conducted using the ‘Complementa’ tool from CAPFITOGEN3 to evaluate the degree of passive protection provided by existing protected areas by assessing species richness inside and outside these areas. It also identified regions with lower medicinal plant representation to inform where conservation initiatives might be expanded [74]. Passive in situ conservation involves minimal intervention within protected areas, meaning that individual species are not specifically managed to maintain their genetic diversity or protect them from pests, diseases, habitat fragmentation, degradation and natural disasters. The assumption is that the overall conservation framework of the protected areas will provide sufficient protection [52,53]. Hotspots of medicinal plant diversity outside of existing protected areas may be localities suitable for new protected area establishment or possible less formally established sites for other effective area-based conservation measures (OECMs) [66].

#### 4.4.2. Ecogeographic-Level Analysis

To identify conservation gaps at the ecogeographical level, as a proxy for genetic diversity, the Representa tool in CAPFITOGEN3 was used to assess the representativeness of ELC zones within protected areas. This tool integrates ELC maps and medicinal plant occurrence records to evaluate how well the ecogeographical diversity of target species is represented within existing protected areas. For the analysis, the ELC map was overlaid with the protected areas map of the Kingdom of Saudi Arabia, and the Representa tool from CAPFITOGEN3 was used to evaluate the representation of ELC zones within existing protected areas. This allowed the identification of ELC zones with low numbers of protected areas, highlighting possible ecogeographical conservation gaps.

### 4.5. Complementarity Analysis

Complementarity analysis is a biodiversity conservation method that has been used extensively since the 1970s [62]. This approach identifies the minimum number of sites needed to conserve the maximum species or taxon diversity, making it an essential tool for prioritising conservation efforts [75,76]. To identify potential sites for the active in situ conservation of medicinal plants in the Kingdom of Saudi Arabia, the ‘Complementa’ tool within CAPFITOGEN3 was used. The analysis was conducted in two steps. First, Complementa was applied to determine the minimum number of existing protected areas needed to conserve the greatest possible diversity of medicinal plants, including both taxon and ecogeographic diversity. Second, for the diversity not already represented within protected areas, Complementa was run again to identify additional 5 × 5 km grid cells outside protected areas that could serve as priority sites for establishing other effective area-based conservation measures or other informal conservation areas. All analyses were conducted using a 5 × 5 km grid resolution (approximately 2.5 arc minutes). This analysis also identified regions where additional active in situ conservation measures are needed, particularly areas with high and unique medicinal plant diversity. For protected areas with similar numbers of unique species, random selection was applied to prioritise the complementary sites for conservation. These priority sites can be located within existing protected areas, designated other effective area-based conservation measures, or other managed sites outside current protected areas [71]. These maps were visualised in CAPFITOGEN3 and QGIS version 3.40 [73].

### 4.6. Ex Situ Conservation Gap Analysis

Ex situ conservation gap analysis is a method used to identify species genetic diversity or ecogeographic diversity that are underrepresented or absent from conservation facilities such as genebanks [33,72].

To identify conservation gaps at the taxon level, the conservation gaps were identified by comparing the distribution of the ex situ accession map with all available occurrence records in the Kingdom of Saudi Arabia map. Ex situ records were compiled from publicly available global repositories, including the Millennium Seed Bank, the Genesys Global Portal and GBIF. These platforms provide international genebank data and known accessions but may not fully capture undocumented or non-digitized holdings in national or smaller institutional collections (e.g., botanical gardens). Therefore, the results reflect gaps in accessible global data rather than a complete inventory of all possible ex situ conservation efforts. This comparison enables the identification of areas where ex situ conservation is currently lacking. To identify conservation gaps at the ecogeographical level, the ‘Representa’ tool from CAPFITOGEN3 was used to categorise the ecogeographic representativeness of existing ex situ collections. The frequency distribution of ELC zones represented in ex situ accessions was compared against the overall ecogeographic diversity found in all occurrence records [62,77]. Resulting maps were visualised using CAPFITOGEN3 and QGIS version 3.40 [73] at a resolution of 5 × 5 km (approximately 2.5 arc minutes). The ELC map was categorised into four classes (low, mid-low, mid-high and high) based on the number of ex situ accessions collected from each ELC zone, thereby indicating the degree of representation of each ecogeographic zone in ex situ conservation. Zones where no occurrence records were found were labelled as ‘Null’ [62,77].

## 5. Conclusions

This study identified critical conservation gaps in both in situ and ex situ efforts for priority medicinal plants in the Kingdom of Saudi Arabia. While the establishment of several protected areas has improved in situ conservation, many species remain passively protected and are underrepresented, particularly in ecogeographically diverse regions. For instance, more than half of the 66 species recorded within protected areas lack targeted management plans or active conservation actions. Complementarity analysis underscored the need to expand conservation actions in areas not currently covered by protected areas and to enhance the protection of adaptive diversity. The ex situ analysis revealed a significant shortage of both taxon and ecogeographic representation in genebanks, with minimal duplication at international facilities and an absence of active national genebanks. To address these challenges, an integrated conservation approach is essential, combining habitat protection, targeted collecting and improved ex situ representation. Building technical capacity in areas such as GIS, biotechnology and community engagement will be critical to support these efforts. Developing a ‘Conservation Strategy and Action Plan for medicinal plants of the Kingdom of Saudi Arabia’ will provide a comprehensive framework to guide future conservation actions, strengthen collaboration between in situ and ex situ efforts and ensure the long-term preservation and sustainable use of medicinal plant genetic resources.

## Figures and Tables

**Table 1:** The complementary protected areas (PAs) for the in situ conservation of medicinal plant species (MPs) in the Kingdom of Saudi Arabia.

Rank	Complementary PAs	No. of Observation Records	No. of Unique MPs	Province
1	Imam Faisal bin Turki Royal Reserve	133	35	Asir, Jazan, and Makkah
2	King Salman Bin Abdalaziz Royal Natural Reserve	25	11	Hail, Tabuk, Al-Jowf, and Northern Borders
3	Prince Mohammed bin Salman Royal Reserve	32	4	Tabuk
4	Jabal Shada al-A‘la	19	3	Al-Baha
5	Jabal al-Lawz	8	3	Tabuk
6	Asir National Park	6	1	Asir
7	Tuwaiq Reserve	14	1	Riyadh
8	Jabal Batharah/Wadi Turabah	10	1	Makkah
9	Jabal al-Dubbagh	2	1	Tabuk
10	Juzur Farasan	8	1	Jazan
11	Jabal Radwa	8	1	Medina
12	Al-Taif National Park/Hima Saysad	1	1	Makkah
13	Wadi Jawwah/Wadi Jazan	2	1	Jazan

**Table 2:** Summary of recommended conservation actions for medicinal plant species in the Kingdom of Saudi Arabia.

Recommendation	Purpose/Outcome
1. Improve cooperation between in situ and ex situ medicinal plant conservation programs and increase government support	Strengthen coordination, funding and community involvement (e.g., citizen science)
2. Ground truth medicinal plant presence in protected areas and develop a national conservation strategy and action plan	Validate species presence and create a strategic framework with threat assessments
3. Expand small protected areas or utilize other effective area-based conservation measures in priority medicinal plant areas	Increase in situ coverage in biodiversity hotspots (e.g., Asir and Jazan)
4. Conduct targeted field surveys for underrepresented medicinal plant species	Ensure representation of medicinal plant species in at least five protected areas as a minimum conservation threshold (Tables S7 and S8) [69]
5. Enhance genebanks: facilities, training and field collection	Improve ex situ resources, genetic monitoring and redundancy (e.g., Svalbard Seed Vault)
6. Apply genomic and biotechnology tools	Support propagation via tissue culture, cryopreservation and genetic conservation
7. Analyse climate change impacts	Anticipate distribution shifts and integrate findings into future conservation planning
8. Employ GIS and remote sensing tools	Support habitat mapping and dynamic monitoring of medicinal plant distributions
9. Strengthen international partnerships and legal frameworks	Increase knowledge exchange, capacity building and regulatory support
10. Review and update conservation priorities	Maintain alignment with latest data and science, emphasizing endemic/traditionally used species
11. Document ethnopharmacological uses of vulnerable medicinal plant species	Integrate traditional knowledge into conservation prioritization and awareness
12. Ensure practical application of recommendations	Translate strategies into national plans, regional actions and local community engagement

## Data Availability

The authors confirm that the data supporting the findings of this study are available within the article.

## Supplementary Materials

The following supporting information can be downloaded at https://www.mdpi.com/article/10.3390/plants14172629/s1.
